# Retrospective study to identify homocysteine reference intervals in healthy Chinese 60 years of age and above

**DOI:** 10.5937/jomb0-40154

**Published:** 2023-10-27

**Authors:** Jianmin Zong, Yue Sun

**Affiliations:** 1 Nanjing Prevention and Treatment Center for Occupational Diseases, Department of Laboratory Medicine, Nanjing, China; 2 Lianyungang Hospital Affiliated to Nanjing University of Chinese Medicine, Department of Laboratory Medicine, Lianyungang, China

**Keywords:** homocysteine, reference intervals, indirect methods, aged, homocistein, referentne vrednosti, indirektne metode, starenje

## Abstract

**Background:**

Homocysteine (Hcy) are associated with many age-related diseases. Heterogeneous physiology with aging combined with unresolved assays standardization necessitates the establishment of specific Hcy reference intervals (RIs) applicable to the elderly. This retrospective study aimed to identify Hcy RIs in the elderly aged 60 years and older from a hospital in Jiangsu Province, China.

**Methods:**

Data from individuals undergoing routine physical examinations were collected. Hcy were measured on Hitachi 7600 analyzer using hydrolase-based enzymatic cycling method. Outliers were identified by Dixon methods. Age- and gender-specific differences were estimated by nonparametric tests. Factors affected Hcy were assessed using multivariate linear regression. RIs with 90% confidence intervals were determined by nonparametric method.

**Results:**

A total of 2594 individuals were included. Hcy levels increased with age (r=0.248, p<0.001). Males have consistently higher Hcy levels (median (interquartile range): 11.95 (8.89-15.30) mmol/L) than females (9.65 (7.05-12.69) mmol/L; p<0.001). Multivariate adjustment analysis showed correlations between Hcy and gender (b=0.188, p<0.001), age (b=0.427, p<0.001) were significant. The Hcy RIs were 5.10-25.46 mmol/L for males, and 4.14-18.91 mmol/L for females, respectively.

**Conclusions:**

This study identified ageand gender-specific Hcy RIs in the elderly, which may guide clinicians in interpreting laboratory findings and clinical management.

## Abstract (sr)

**Uvod:**

Homocistein (Hcy) je povezan sa mnogim bolestima vezanim za uzrast. Heterogena fiziologija sa starenjem u kombinaciji sa nerazjašnjenom standardizacijom testova zahteva uspostavljanje specifičnih Hcy referentnih intervala (RI) primenljivih na starije osobe. Ova retrospektivna studija imala je za cilj da identifikuje Hcy RI starosti 60 godina i više iz bolnice u provinciji Jiangsu, Kina.

**Metode:**

Prikupljeni su podaci od pojedinaca koji su bili podvrgnuti rutinskim fizičkim pregledima. Hcy je izmeren na Hitachi 7600 analizatoru koriščenjem metode enzimskog ciklusa zasnovanog na hidrolazi. Izuzetne vrednosti su identifikovane Diksonovim metodama. Razlike po uzrastu i polu su procenjene neparametarskim testovima. Faktori koji utiču na Hcy su procenjeni korišćenjem multivarijantne linearne regresije. RI sa 90% intervala poverenja određeni su neparametrijskom metodom.

**Rezultati:**

Uključeno je ukupno 2594 osobe. Nivo Hcy se povećavao sa godinama (r=0,248, p<0,001). Muškarci imaju konstantno više Hcy nivoe (medijan (interkvartilni opseg): 11,95 (8,89-15,30) pmol/L) od žena (9,65 (7,05-12,69) mmol/L; p<0,001). Multivarijantna analiza prilagođavanja pokazala je da su korelacije između Hcy i pola (b=0,188, p<0,001), i starosti (b =0,427, p<0,001) bile značajne. Hcy RI su bili 5,10-25,46 pmol/L za muškarce, odnosno 4,14-18,91 pmol/L za žene.

**Zaključak:**

Ova studija je identifikovala Hcy RI specifične za uzrast i pol kod starijih osoba, što može poslužiti kliničarima u tumačenju laboratorijskih nalaza i kliničkom upravljanju.

## Introduction

Homocysteine (Hcy) has been reported to be associated with many age-related diseases such as cardio-cerebrovascular diseases, neurodegenerative diseases, cognitive impairment, osteoporotic fractures, and even cancer [1] [2] [3].

China is aging, data from the National Census in 2021 released that people aged 60 and above have reached 264 million, accounting for 18.7% of the total population [4]. Hcy has gaining importance in geriatric healthcare delivery and an appropriate reference interval (RI) is of practical significance to be established.

There is still a gap between Hcy RI establishment and application. To date, standardization of assays remains unresolved due to the lack of certified reference method and material [5], multiple assays such as chromatography, enzymatic assay and immunoassay are used in different laboratories and the variations among results are actually considerable [6]. Regardless of analytical platforms, intervals of 5–15 mmol/L (two-sided) or <15 mmol/L (right-sided) are widely adopted as Hcy RIs in practice [7] [8], which seems to be clinically inappropriate.

Heterogeneous changes accompanied by normal aging result in much wider intervals in the elderlythan in adults and they are not sufficient to be considered a pathological condition, which has been well characterized by previous studies. Since factors such as age, gender, and ethnicity are known to affect Hcy results [9], we suspect the non-partitioned Hcy RI might significantly complicate the interpretation of laboratory findings in the elderly.

Although clinical laboratories have been recommended for decades to establish RIs appropriate to the assay method and local population [10], more than 80% of laboratories in China prefer to adopt RI provided by manufacturer [11], limited studies on RIs establishment are available so far, and the subjects are mainly constricted to adult [12] [13] [14] [15] [16], elderly has not been a detailed description yet due to the limited sample size.

Due to difficulties of recruiting enough elderly healthy individuals, indirect methods were proposed in the Clinical and Laboratory Standards Institute (CLSI) EP28-A3c guideline [10]. In this approach, data were extracted from the database by applying exclusion criteria and statistical methods, rather than collecting samples from recruited subjects, which is relatively feasible for individual laboratories.

Therefore, this retrospective study aimed to analyze the effects of indicators such as gender and age on Hcy and determine the RIs in the elderly aged 60 years and older by indirect method.

## Materials and methods

### Study design

To extract data from relatively healthy subjects, we retrieved data from individuals undergoing routine physical examinations for periodic health screening rather than all outpatients from January 1, 2018 to June 30, 2021. The last available data of the same individual was retained. The following exclusion criteria were applied as extra precautions to remove underlying pathological results may be present in the database. Individuals with missing records will be considered unqualified and their test results will be excluded.

1. Self-reported history or drugs treatment in past history records, including endocrine diseases, autoimmune disease, cardiovascular diseases, respiratory diseases, hematological diseases, cancer.

2. Abnormalities found in ultrasound, electrocardiogram and X-ray examination.

3. Body mass index (BMI) ≥ 28 kg/m^2^ or 18.5 kg/m^2^.

4. Hypertension: systolic blood pressure (SBP) ≥140 mmHg or diastolic blood pressure (DBP) ≥90 mmHg.

5. Hyperlipidemia: total cholesterol (TC) ≥6.22 mmol/L, triglyceride (TG) ≥1.70 mmol/L.

6. Hyperglycemia: fasting blood glucose (FBG) ≥ 7.0 mmol/L.

7. Hemoglobin (HGB)<120 g/L (for males) or 110 g/L (for females); white blood cell (WBC) >13.0×10^9^/L or<3.0×10^9^/L; platelet (PLT) >350×10^9^/L or<100×10^9^/L; high sensitivity C-reaction protein (hsCRP) >10.0 mg/L.

8. Abnormal liver and kidney function: albumin (ALB) <40 g/L; alanine aminotransferase (ALT) >50 U/L (for males) or >40 U/L (for females); creatinine (Crea) >111 mmol/L (for males) or>81 mmol/L (for females); urea>9.5 mmol/L (for males) or>8.8 mmol/L (for females).

9. Positive results of hepatitis B surface antigen (HBsAg), anti-hepatitis C virus (anti-HCV), or antihuman immunodeficiency virus (anti-HIV).

The Ethics Committee of Lianyungang Hospital affiliated to Nanjing University of Traditional Chinese Medicine deemed the study exempt from review. This retrospective study was conducted on anonymizationbased already available data and informed consent was waived.

### Measurements

About 5 mL of venous blood was drawn into gel separator tubes (BD biosciences, New Jersey, USA) after an overnight fast. All samples were collected between 7:00 am and 11:00 am. Samples were centrifuged for 10 minutes at 1,500 g within 1 hour after collection. Lipemic or hemolyzed samples were considered ineligible. Samples were loaded into Hitachi 7600 automatic analyzer (Hitachi Co., Ltd, Tokyo, Japan) within 2 hours, Serum Hcy concentrations were analyzed by hydrolase-based enzymatic cycling method with reagents provided by Beijian Xinchuangyuan (Beijian BJ Biotech Co., Ltd, Beijing, China). The linearity range is 3.0–45.0 mmol/L. Samples will be diluted with normal saline and retested when the detected value of the sample exceeds the linear range. The inter-assay coefficient of variations was 7.85%. Operations were performed according to the standard operating procedures. Internal quality controls were performed prior to every analytical run and deemed to be valid. We also participated in the external quality assessments of the National Center for Clinical Laboratories twice a year to ensure the results were credible.

### Statistics

Statistical analyses were performed on SPSS 19.0 (SPSS Inc., Chicago, USA) and GraphPad PRISM 9.0 for Windows (GraphPad Software, Inc., San Diego, CA, USA). Outliers were identified using Dixon method. Normality of data was tested by Kolmogorov-Smirnov test. Gaussian distributed data were expressed as means and standard deviation, whereas non-Gaussian distributed data were expressed as medians and interquartile range. Spearman’s test was performed to assess the correlation between age and Hcy values. Age- and gender-specific differences were estimated by Mann-Whitney U test and Kruskal-Wallis H test as appropriate. Multivariate linear regression was used to evaluate the effects of sex, age and other analytes on Hcy levels. RIs were determined by a non-parametric method which expressed as 2.5^th^ and 97.5^th^ percentiles with 90% confidence intervals calculated by a bootstrap-resampling procedure. Two-tailed p value<0.05 was considered statistically significant.

## Results

### Baseline characteristics of subjects

2594 individuals of 60–97 years of age, including 1606 males (61.9%) and 988 females (38.1%) were collected. All of them were Han ethnicity. The Mann-Whitney U test showed gender differences with higher blood pressure, WBC, HGB, ALB, ALT, Crea, TG, FBG levels in males and higher PLT in females (Table 1). Data showed a non-Gaussian distribution according to Kolmogorov-Smirnov test, thus nonparametric methods were used.

**Table 1:** Baseline characteristics of included individuals. ^a^Tested with Mann-Whitney U test for variables between genders.<br>Abbreviations: SBP, systolic blood pressure; DBP, diastolic blood pressure; WBC, white blood cell; HGB, hemoglobin; PLT, platelet; hsCRP, high sensitivity C-reaction protein; ALB, albumin; ALT, alanine aminotransferase; Crea, creatinine; TC, total cholesterol; TG, triglycerides; FBG, fasting blood glucose.

Index	Total<br>(n=2594)	Males<br>(n=1606)	Females<br>(n=988)	p value^a^
Age (years)	64(64, 75)	69(64, 76)	67(63, 73)	<0.001
SBP (mmHg)	116(108,126)	120(112,128)	111(103,120)	<0.001
DBP (mmHg)	72(65,78)	75(69,81)	66(61,73)	<0.001
WBC (×10^9^/L)	6.02(5.02,7.18)	6.09(5.18,7.20)	5.93(4.73,7.12)	<0.001
HGB (×10^9^/L)	142(133,152)	148(140,157)	134(127,140)	<0.001
PLT (×10^9^/L)	190(159,226)	180(154,214)	207(173,244)	<0.001
hsCRP (mg/L)	1.00(0.93,2.72)	1.00(0.93,2.91)	1.00(0.90,2.58)	0.286
ALB (g/L)	47.6(46.1,49.2)	47.9(46.4,49.4)	47.3(45.7,48.7)	<0.001
ALT (U/L)	18(13,24)	19(15,26)	16(12,21)	<0.001
Crea (μmol/L)	67(55,78)	75(68,83)	53(48,59)	<0.001
Urea (mmol/L)	4.98(4.21,5.88)	5.27(4.56,6.15)	4.46(3.75,5.29)	<0.001
TC (mmol/L)	4.78(4.19,5.31)	4.77(4.18,5.31)	4.81(4.21,5.34)	0.057
TG (mmol/L)	1.24(0.85,1.64)	1.26(0.89,1.63)	1.21(0.79,1.65)	<0.001
FBG (mmol/L)	5.33(5.05,5.65)	5.38(5.09,5.71)	5.26(5.02,5.56)	<0.001

### Distribution of Hcy levels according to gender and age

**Figure 1:**
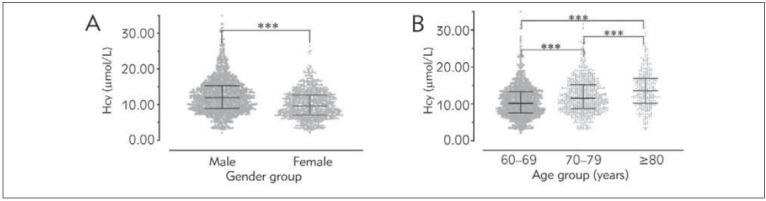
Distribution of Hcy stratified by (A) gender and (B) age. The lines and error bars represent medians and interquartile ranges, respectively. ^***^P<0.001

Males had consistently higher Hcy levels (11.95 (8.89–15.30) mmol/L) than females (9.65 (7.05–12.69) mmol/L; p<0.001). Hcy levels showed steady upward movement in the three randomly defined age groups (60–69, 70–79 and ≥80 years) and significant differences were noted in pairwise comparisons (p<0.001) (Figure 1).

### Effects of confounders on Hcy levels

The multivariate liner regression analysis revealed that Hcy was positively associated with many variables including gender, age, blood pressure, PLT, ALB, ALT, Crea, Urea, TC, TG and FBG (p<0.05). After age- and sex-adjustment, gender (β=0.188, p<0.001), age (β=0.427, p<0.001), SBP (β=0.041, p=0.026), ALB (β=0.046, p=0.048) and TC (β=0.056, p=0.040) remained statistically significant (Table 2).

**Table 2:** Multivariate linear regression of the association between various factors with Hcy. Unstandardized coefficients were expressed as B (B-coefficient) and SE (standard error); standardized coefficients were expressed as Beta.<br>^a^Coefficients are calculated to three decimal figures.<br>Abbreviations: Hcy, homocysteine; SBP, systolic blood pressure; DBP, diastolic blood pressure; WBC, white blood cell; HGB, hemoglobin; PLT, platelet; ALB, albumin; ALT, alanine aminotransferase; Crea, creatinine; TC, total cholesterol; TG, triglycerides; FBG, fasting blood glucose.

Factors	Unadjusted			Age- and sex-adjusted		
	B (SE)	Beta	p value	B (SE)	Beta	p value
Gender<br>(0: female 1: male)	2.477 (0.194)	0.243	<0.001	1.915 (0.176)	0.188	<0.001
Age (years)	0.307 (0.012)	0.452	<0.001	0.291 (0.012)	0.427	<0.001
SBP (mmHg)	0.061 (0.008)	0.147	<0.001	0.017 (0.008)	0.041	0.026
DBP (mmHg)	0.083 (0.011)	0.150	<0.001	0.010 (0.010)	0.018	0.343
WBC (×10^9^/L)	0.103 (0.060)	0.034	0.086	-0.017 (0.053)	-0.005	0.751
HGB (×10^9^/L)	0.001(0.007)	0.003	0.867	-0.015(0.007)	-0.041	0.404
PLT(×10^9^/L)	-0.009 (0.002)	-0.088	<0.001	0.000^a ^(0.002)	0.000^a^	0.998
ALB (g/L)	0.162 (0.041)	0.077	<0.001	0.097 (0.036)	0.046	0.048
ALT (U/L)	0.051 (0.012)	0.086	<0.001	0.022 (0.010)	0.037	0.056
Crea (μmol/L)	0.061 (0.006)	0.190	<0.001	-0.007 (0.008)	-0.022	0.370
Urea (mmol/L)	0.322 (0.076)	0.083	<0.001	-0.095 (0.070)	-0.024	0.179
TC (mmol/L)	0.175 (0.121)	0.029	0.146	0.135 (0.105)	0.056	0.040
TG (mmol/L)	0.137 (0.238)	0.011	0.564	-0.003 (0.208)	0.000^a^	0.989
FBG (mmol/L)	0.752(0.192)	0.077	<0.001	0.199(0.169)	0.020	0.239

### Established Hcy RIs in the elderly

**Figure 2:**
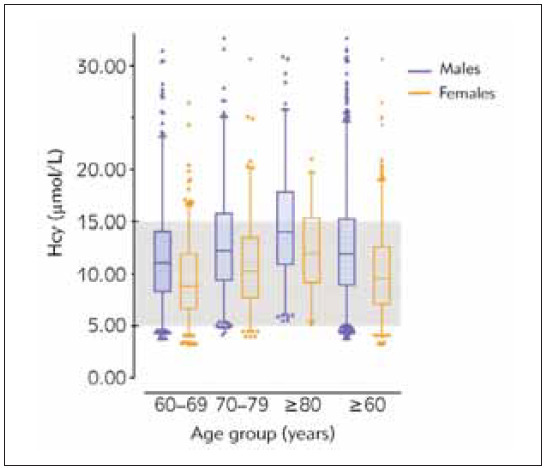
Hcy reference intervals in males and females 60 years of age and above. The boxplot describes the medians (lines in the boxes), 25^th^–75^th^ percentiles (limits of boxes), and 2.5^th^–97.5^th^ percentiles (vertical lines) of Hcy concentrations. Grey area represents the currently recognized reference interval (5–15 μmol/L).

Based on the results above, we resulted in gender- and age-specific RIs in the elderly (Table 3). And due to the overlap of 90% confidence intervals of the upper and lower limit, RIs for males and females combining the different age groups were calculated as well. Gender- and age-partitioned Hcy RIs compared to those of manufactures are shown in Figure 2.

**Table 3:** Gender- and age-specific Hcy reference intervals (μmol/L). Abbreviations: Hcy, homocysteine; LL, lower limit; UL, upper limit; CI, confidence interval.

Age,<br>years	Male Reference intervals				Female Reference intervals			
	Sample,n	Median	LL (90%CI)	UL (90%CI)	Sample,<br>n	Median	LL (90%CI)	UL (90%CI)
60–69	836	11.11	4.66 (4.01–5.06)	23.37 (22.66–25.72)	613	8.86	4.10 (4.06–4.16)	16.55 (15.45–16.96)
70–79	523	12.26	5.42 (5.08–5.69)	26.69 (24.05–31.30)	283	10.28	4.51 (4.47–5.02)	20.16 (18.99–24.86)
≥80	247	14.04	6.09 (5.90–6.35)	25.77 (24.60–29.10)	92	12.00	5.47 (5.12–6.02)	19.66 (18.91–20.97)
≥60	1606	11.95	5.10 (4.87–5.28)	25.46 (24.00–26.69)	988	9.65	4.14 (4.09–4.30)	18.91 (18.02–19.36)

### Comparison of Hcy RIs across previous studies

The Hcy RIs reported across studies showed considerable differences (Table 4). Most studies resulted in gender- partitioned RIs; some of them also provided RIs by different age subgroups.

**Table 4:** Hcy reference intervals determined in previous studies (μmol/L). Values are presented as the medians (2.5^th^–97.5^th^ percentiles) unless otherwise indicated.<br>^a^ Values are presented as the medians (5^th^–95^th^ percentiles); ^b^ Values are presented as the means (2.5^th^–97.5^th^ percentiles)<br>Abbreviations: Hcy, homocysteine; M, male; F, female; LC-MS/MS: liquid chromatography-tandem mass spectrometry; RS, reflectance spectrophotometry; FPIA, fluorescence polarization immunoassay; HPLC, high performance liquid chromatography.

Author	Year	Source	Assay	Age,<br>years	Sample size,<br>n	Reference Interval
Si et al. [17]	2022	Chinese	Enzymatic assay	61–90	M:8570<br>F: 2886	14.70 (10.00–20.90)<br>12.40 (8.40–16.30)
Cui et al. [12]	2022	Chinese	LC-MS/MS	21–79	M:710<br>F: 843	10.66 (6.09–17.00)<br>8.19 (4.61–14.61)
Adeli et al. [13]	2015	Canadian	Enzymatic/RS	26–79	M:680<br>F: 420	8.30 (5.20–14.10)<br>6.90 (3.70–10.90)
Lahiri et al. [14]	2014	Indian	Enzymatic assay	20–81	M:636<br>F: 652	11.44 (6.50–16.38)
Kweon et al. [18]	2014	South Korean	FPIA	65–74	M:414<br>F: 630	7.93 (5.12–13.90)^a^<br>6.47 (3.95–10.65)^a^
Moon et al. [15]	2011	South Korean Hcy	FPIA	21–65	M:220<br>F: 160	11.59 (7.26–19.21)^b^<br>8.50 (5.50–14.99)^b^
Taskin et al. [16]	2006	Turk	HPLC	22–81	M:118<br>F: 41	9.52 (5.57–20.55)^b^<br>7.39 (4.40–16.20)^b^
Ganji et al. [19]	2006	American<br>(non-Hispanic white)<br>American<br>(non-Hispanic black)<br>American (Hispanic)	FPIA<br>FPIA<br>FPIA	>70<br>>70<br>>70	M:510<br>F: 515<br>M:84<br>F: 114<br>M:142<br>F: 143	10.80 (7.28–19.52)^a^<br>9.53(6.15–19.05)^a^<br>11.16(7.02–21.74)^a^<br>10.73 (6.67–26.19)^a^<br>11.19 (7.19–19.12)^a^<br>10.63 (6.39–17.71)^a^
Molero et al. [20]	2006	Venezuelan (Hispanic)	FPIA	>55	M:601<br>F: 302	12.60 (7.43–23.52)<br>11.00 (6.02–20.46)

## Discussion

In the present study, we extracted data from the database from a hospital in Jiangsu Province, China by applying exclusion criteria and statistical methods and identified Hcy RIs in 2594 healthy individuals 60 years of age and above. Higher than the current RI (5–15 mmol/L), RIs established this study were 5.10–25.46 mmol/L for males, and 4.14–18.91 mmol/L for females, respectively.

The findings proved elderly yield higher Hcy values as well as wider ranges than adults. Decreasedrenal function, losses of muscle mass and reduced physical activity in the elderly are reported as determinants [21] [22]. Absorption dysfunction accompanied by aging also put the elderly at higher risk of folate and vitamin B12 deficiencies. Males have higher Hcy levels than females. Although gender differences have been reported by many studies, findings are inconsistent in the elderly. Fonseca et al. [23] found that differences diminish after menopause. No significant association of estradiol and Hcy levels in elderly men was observed by Nakhai et al. [24]. However, our finding of significantly lower Hcy levels in postmenopausal women compared to men were consistent with Cohen et al. [25], suggesting the differences between gender with regard to Hcy were only partially explained by the Hcy-lowering effect of estrogen [26], more determinants need to be taken into account. Gender differences exist in folate and vitamin B12 levels. A NHANES survey indicated that elderly males are more likely to be deficient in these vitamins than females [27]. A prospective study in healthy Swiss seniors also found significantly lower levels of these vitamins in males [28]. Males tend to produce more creatine due to more muscle mass, which connected with methyl transfer, leading to Hcy formation [29]. Additionally, females are less possible exposed to smoke and alcohol consumption, which could influence lowering Hcy levels [30].

Geographical and ethnic differences are significant. Hcy levels were approximately 13 mmol/L in China [17] wheareas in some countries the values were even less than 10 mmol/L [13] [16] [18]. Chinese tend to yield higher Hcy concentrations, which could be ascribed to the gene-environment interaction of a higher prevalence of methylenetetrahydrofolate reductase C677T (MTHFR C677T) mutation [31] and a dietary with no or partial folic acid fortification [32].

Moreover, results could be more comparable despite bias with those of other studies based on Chinese or other closely related ethnicities. In Si et al.’s study [17], Hcy RIs in 60–90 years were 10.00–20.90 mmol/L for males and 8.40–16.30 mmol/L for females respectively. A Korean study showed that the upper limits of RIs for male and female were 19.21 mmol/L and 14.99 mmol/L [15]. The right-sided RIs determined by Ma et al. [33] were 15.80 mmol/L for males and 13.60 mmol/L for females, respectively. These lower values may be due to the fact that their subjects included people aged 18–60 years old, and the latter also determined upper limits from the 95th percentiles rather than the 97.5th percentiles.

Except age and gender, the multivariate-adjusted analysis showed SBP, ALB and TC was also correlated to Hcy concentrations. Despite the statistical significance, the slopes of regression equations are so small that small changes in Hcy require larger changes in these variables. It is worth mentioning, however, that the correlation between blood pressure and Hcy was not as strong as expected, which we speculate is due to the relatively lower blood pressure of the subjects, thus weakening the significance. Data in this study were extracted in normotensive subjects after exclusion of abnormal results. Additionally, the effect of large arterial stiffness in the elderly could result in downward movement of blood pressure to some extent [34]. It also might be related to the fact that people undergoing physical examinations were required to go through an overnight fast and have their blood pressures measured at rest.

There are also some limitations that need to be mentioned. Data in this study were extracted from the database rather than volunteer recruitment. Information including smoke, alcohol consumptionand vitamin supplementation were not available in the database. Given the limitations of approaches, the findings are supposed to be informative rough estimates. Besides, standardization of Hcy assay remains unresolved, variation exists among platforms and reagents. RIs based on enzymatic assays cannot be expanded to other methods.

## Conclusion

The findings from this study identified age- and gender-related Hcy RIs in the healthy population 60 years of age and above in Jiangsu Province, China, which may provide personalized tools in interpreting laboratory findings and clinical management of this patient population.

## Dodatak

### Conflict of interest statement

All the authors declare that they have no conflict of interest in this work.
